# Supplementary figures and images for: Engineered Biological Neural Networks on High Density CMOS Microelectrode Arrays
Source: Front Neurosci. 2022 Feb 21;16:829884. doi: 10.3389/fnins.2022.829884 (PMC8900719; doi:10.3389/fnins.2022.829884)

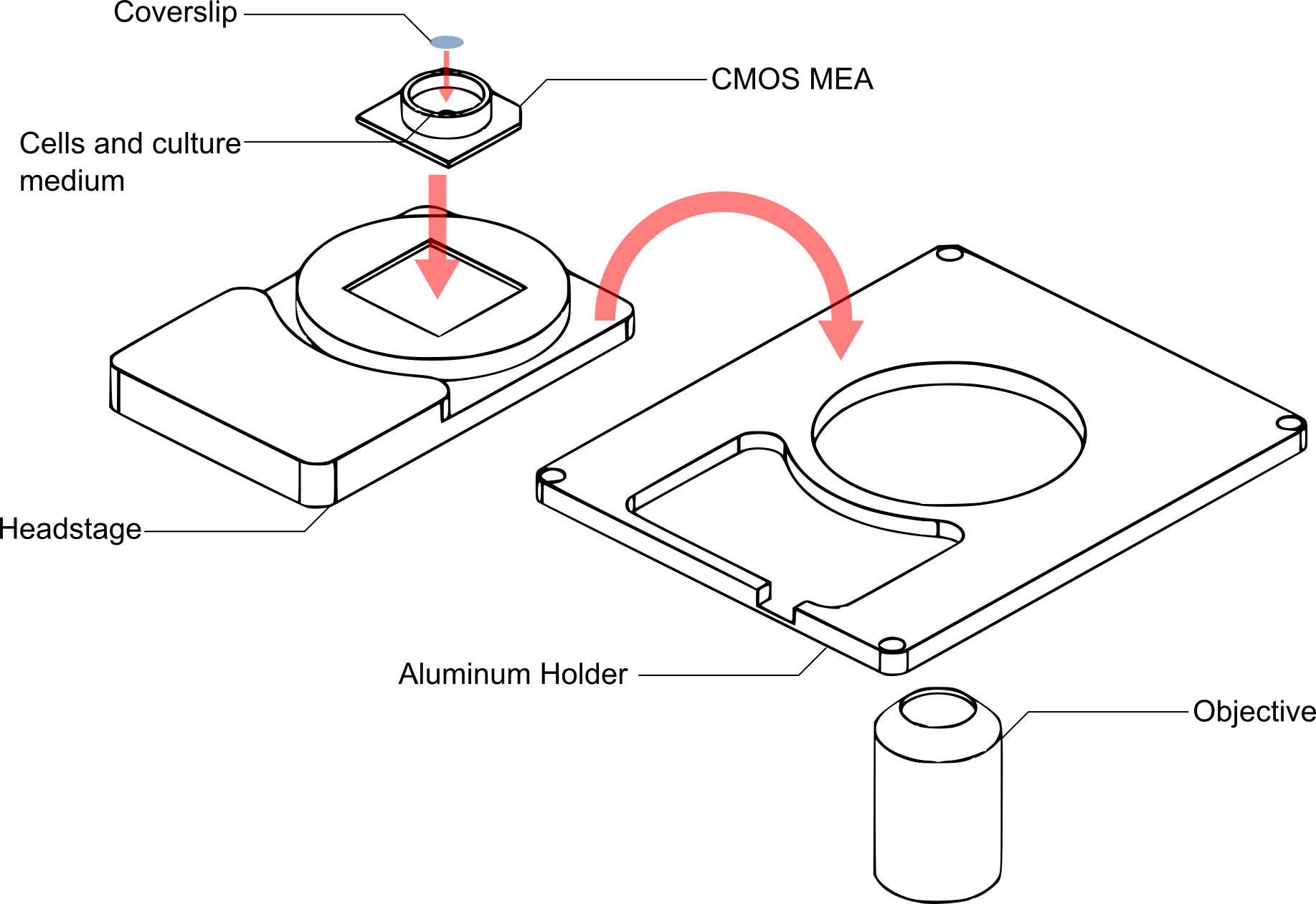

Supplement: Supplementary Image 1 — Procedure of imaging the engineered neural networks growing on the CMOS MEA. A coverslip is placed in the culture medium, which is then carefully aspirated, leaving a thin film of culture medium on top of the culture, secured by the coverslip. The MEA is then placed in the headstage, which is flipped and placed in a custom aluminum holder, that can be inserted in an inverted microscope. [file Image_1.JPEG]

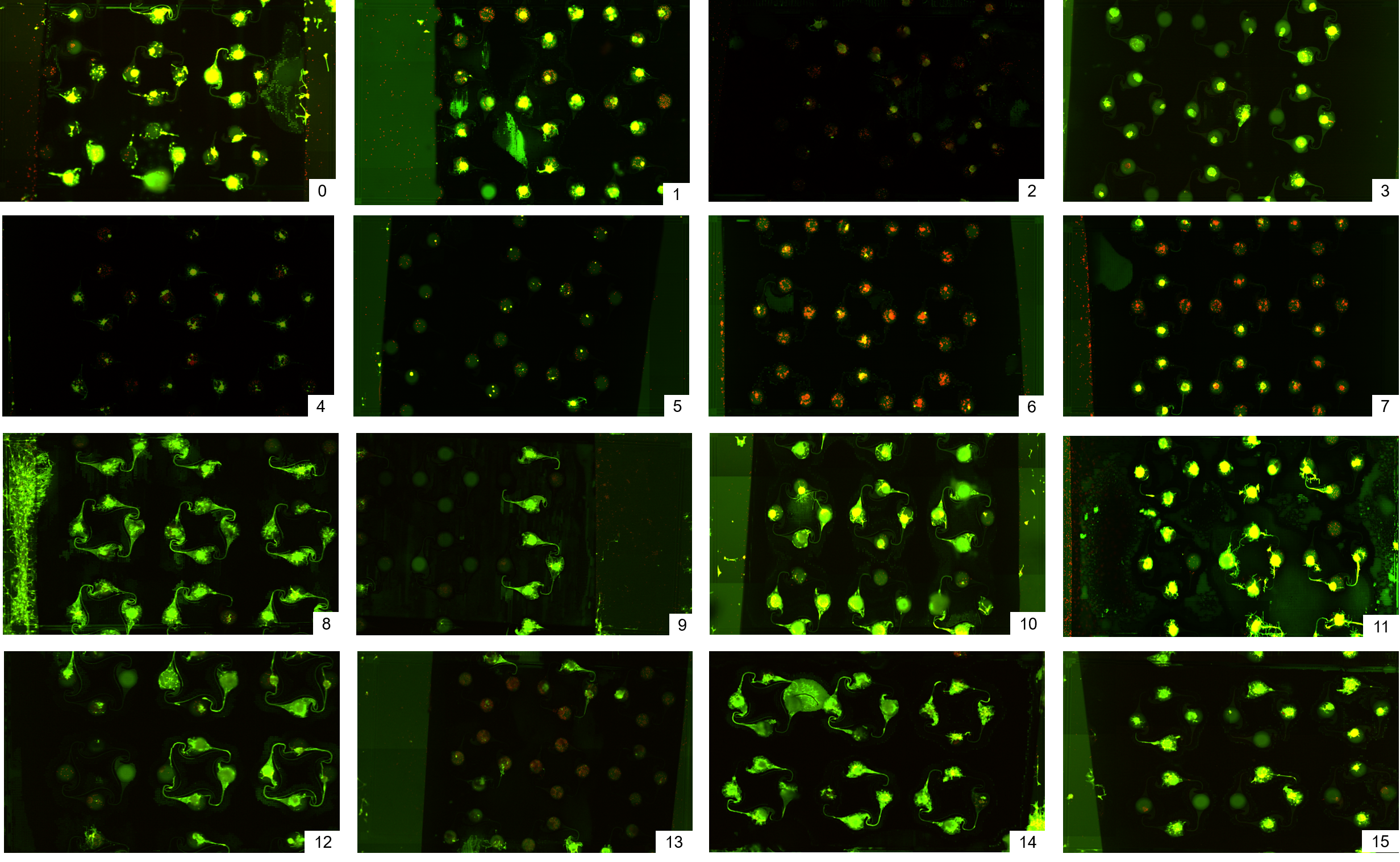

Supplement: Supplementary Image 2 — Live-dead staining using CMFDA (green, alive) and ethidium homodimer (red, dead) on all 16 CMOS MEAs. [file Image_2.JPEG]

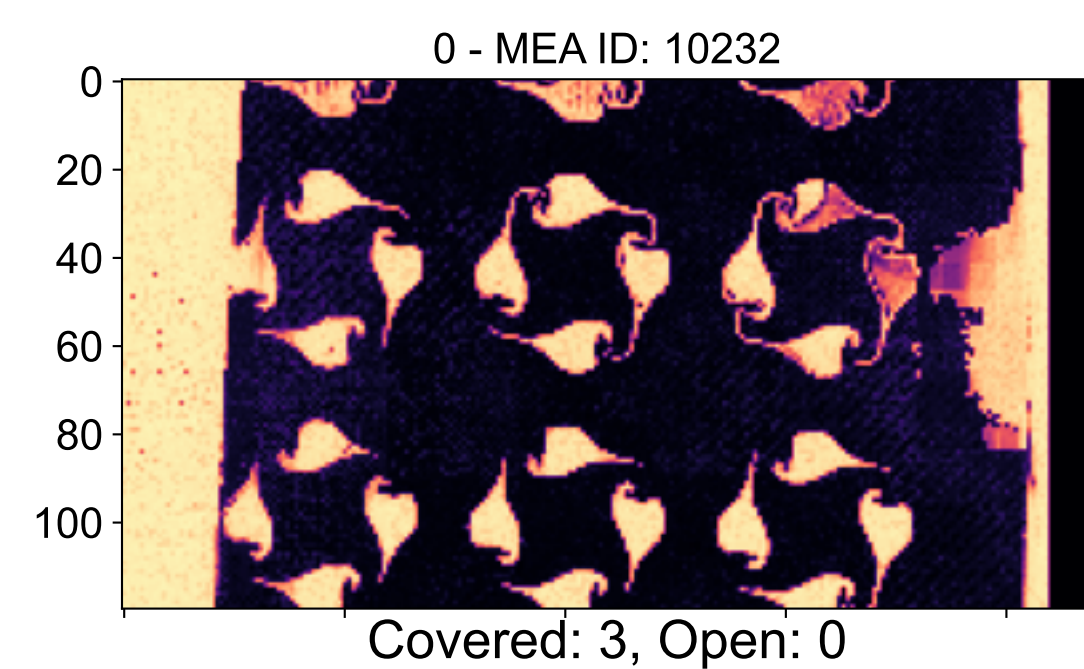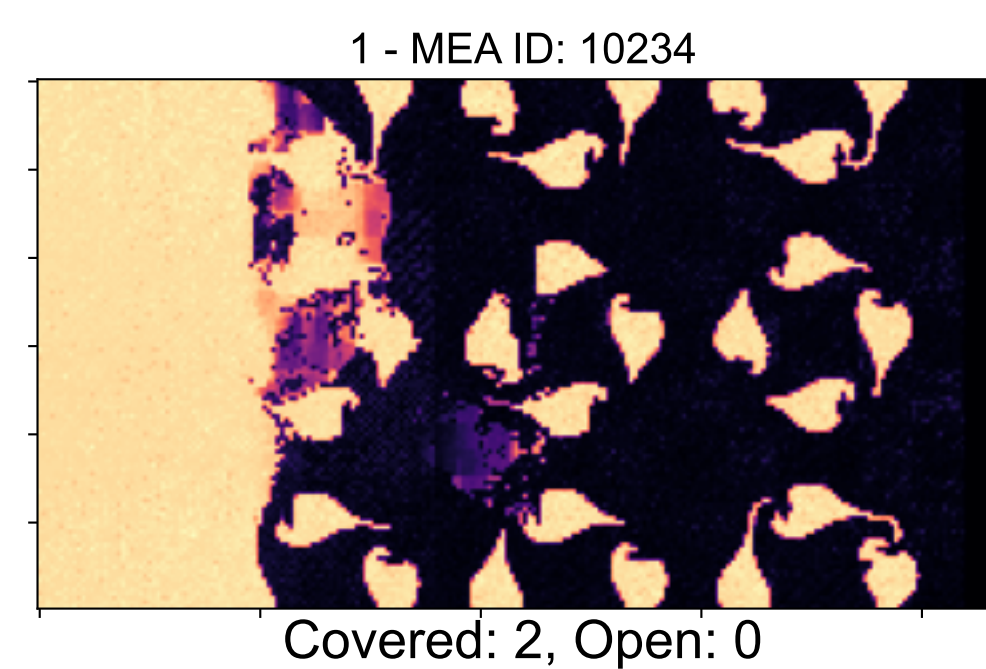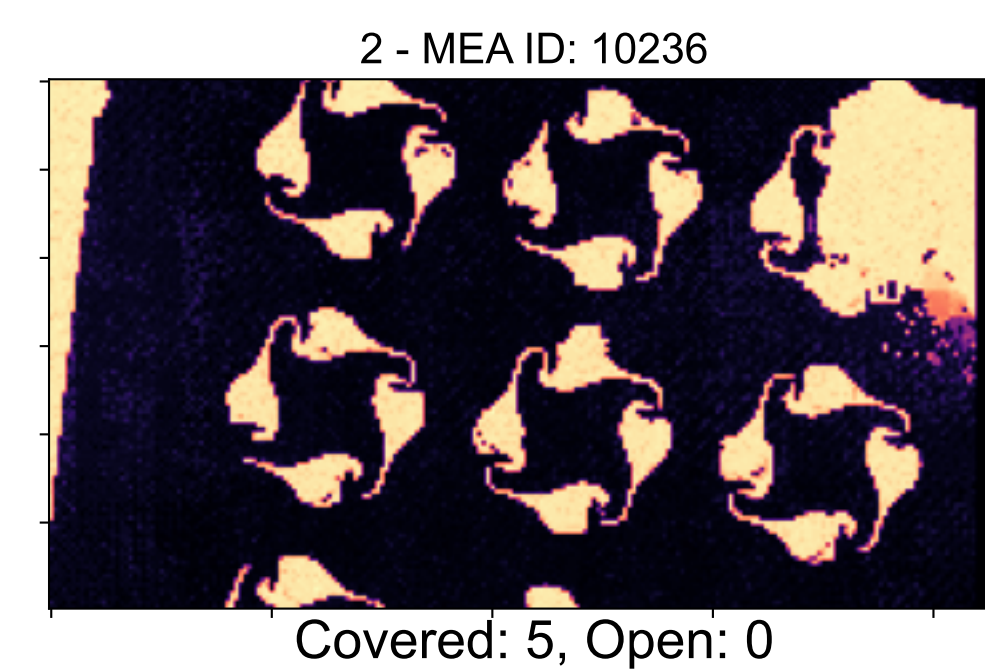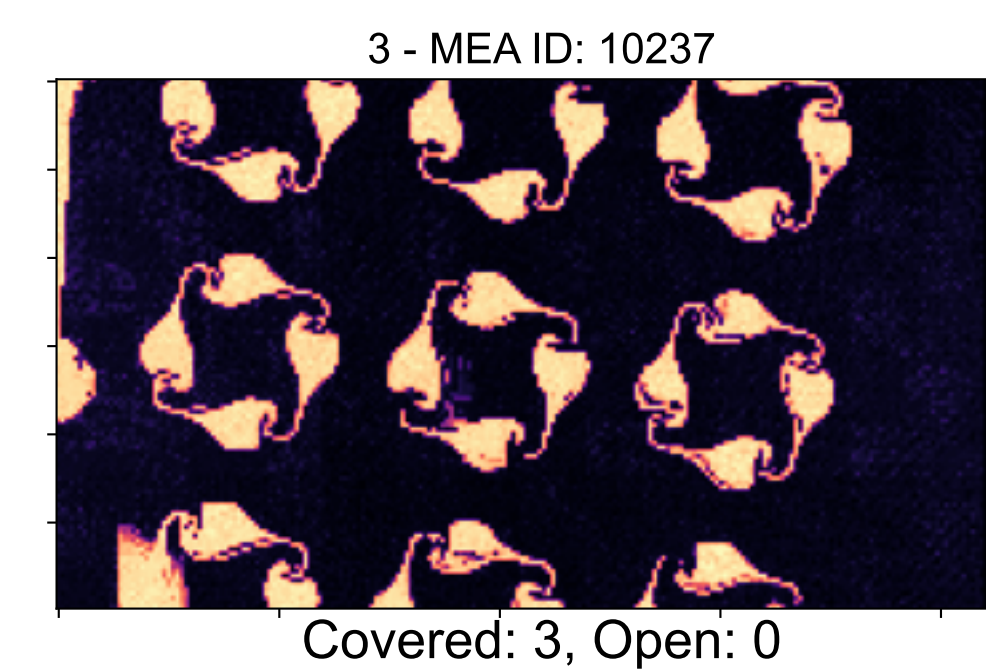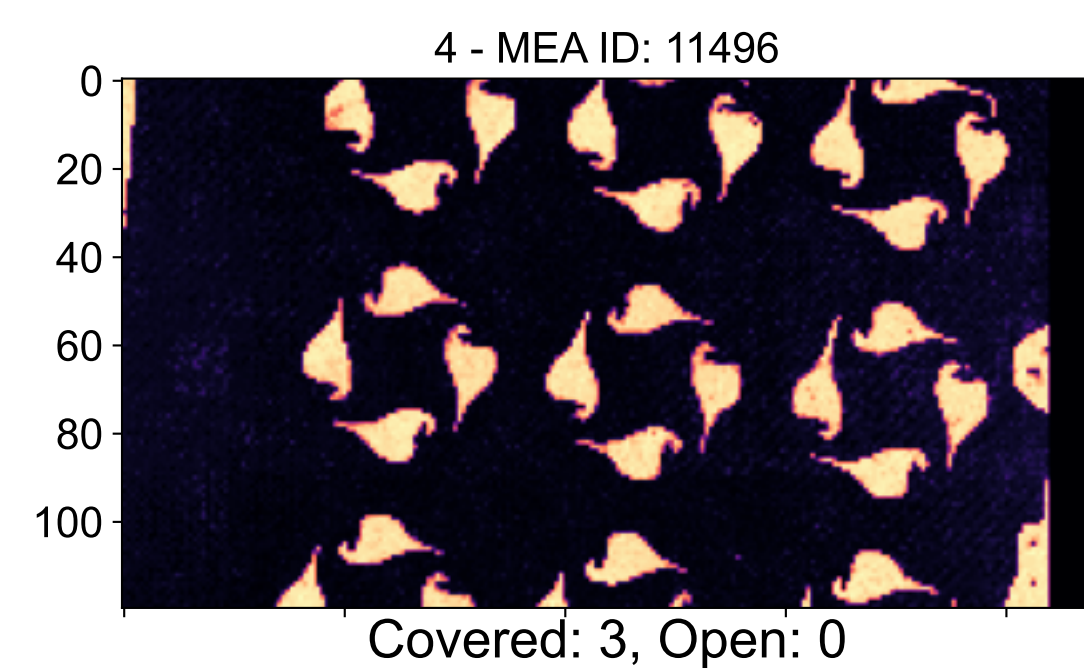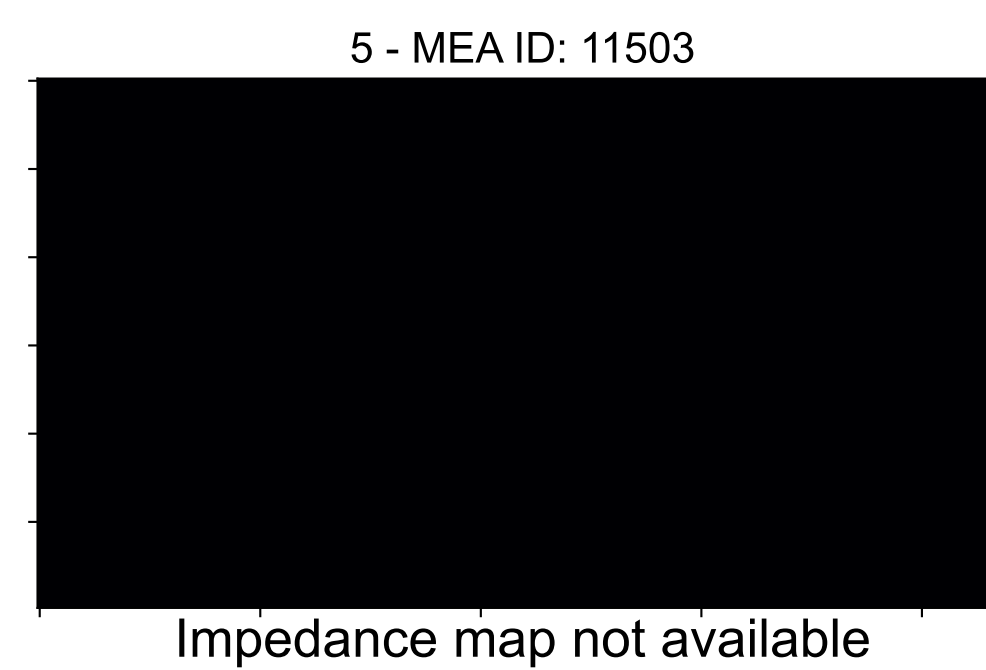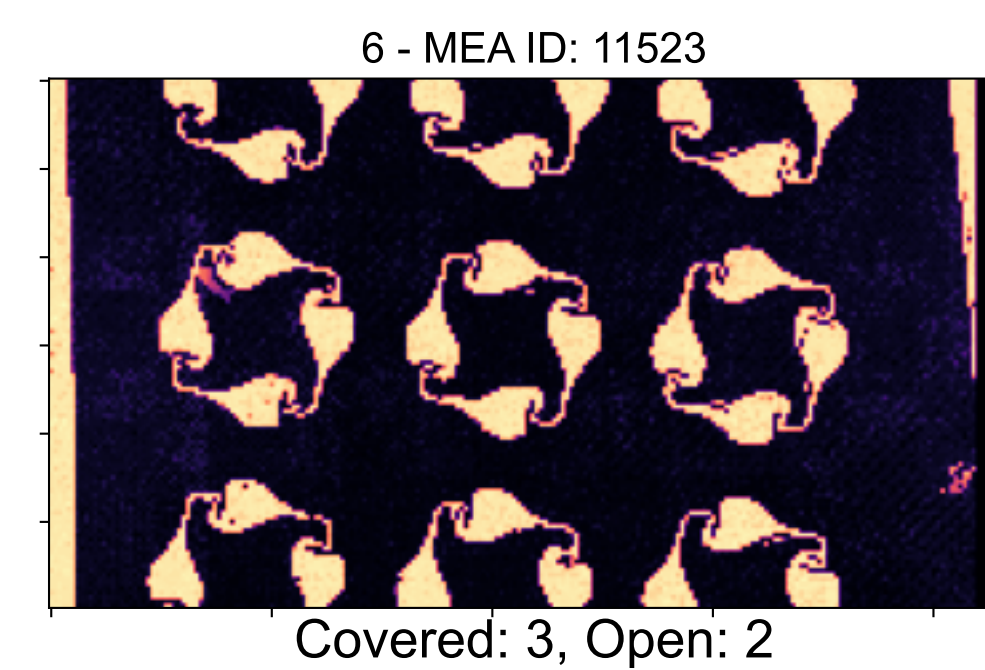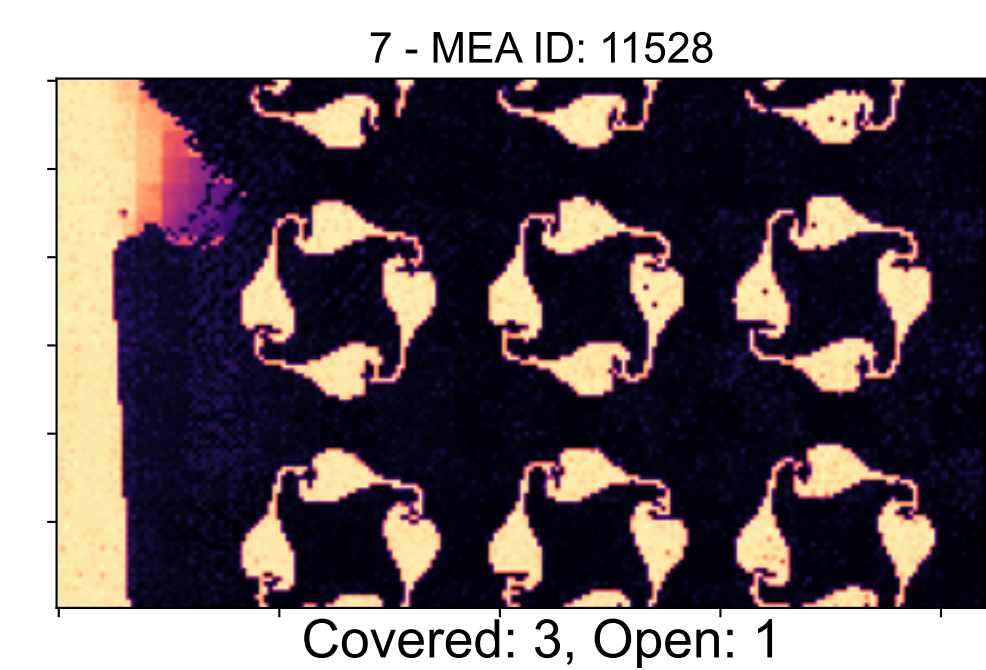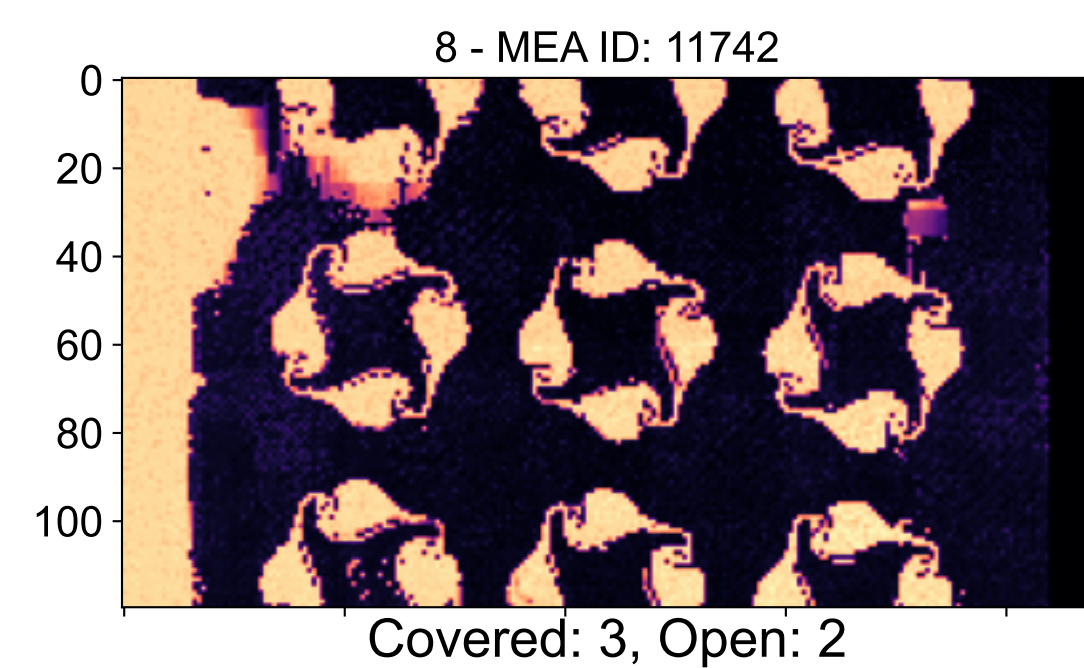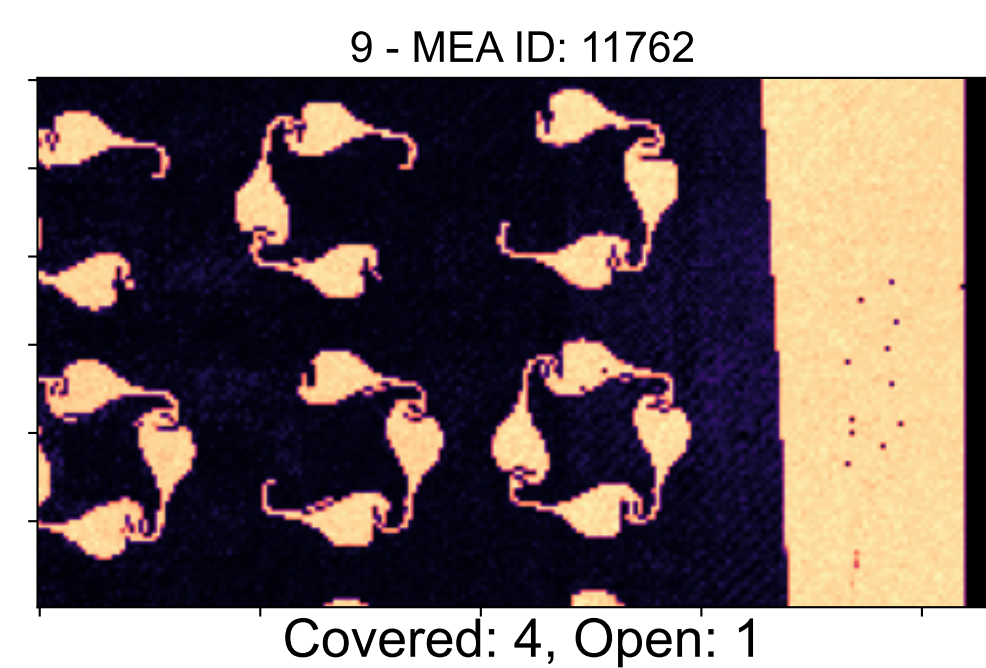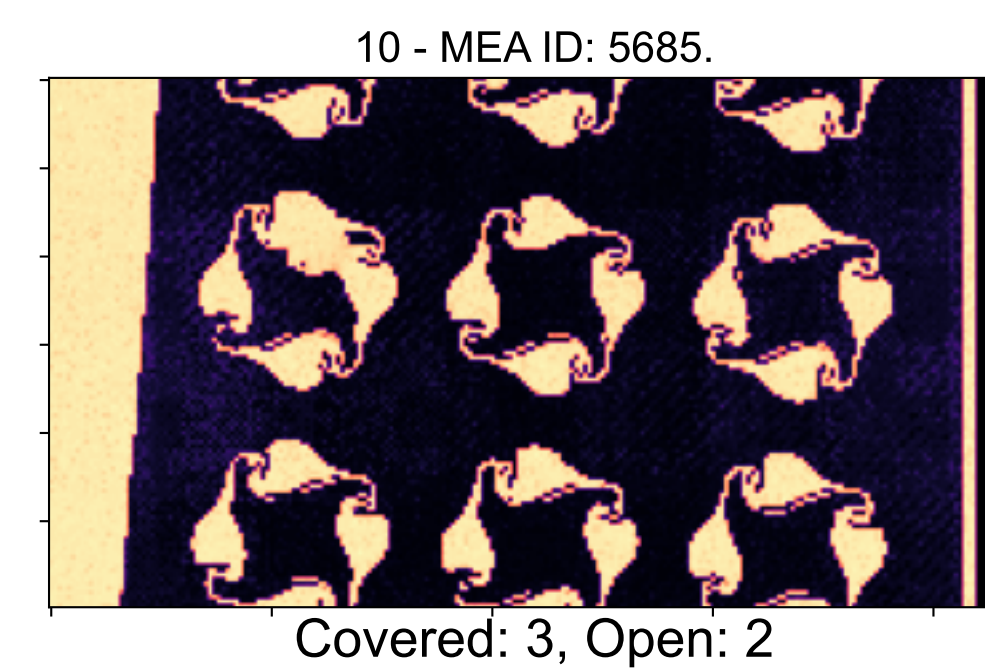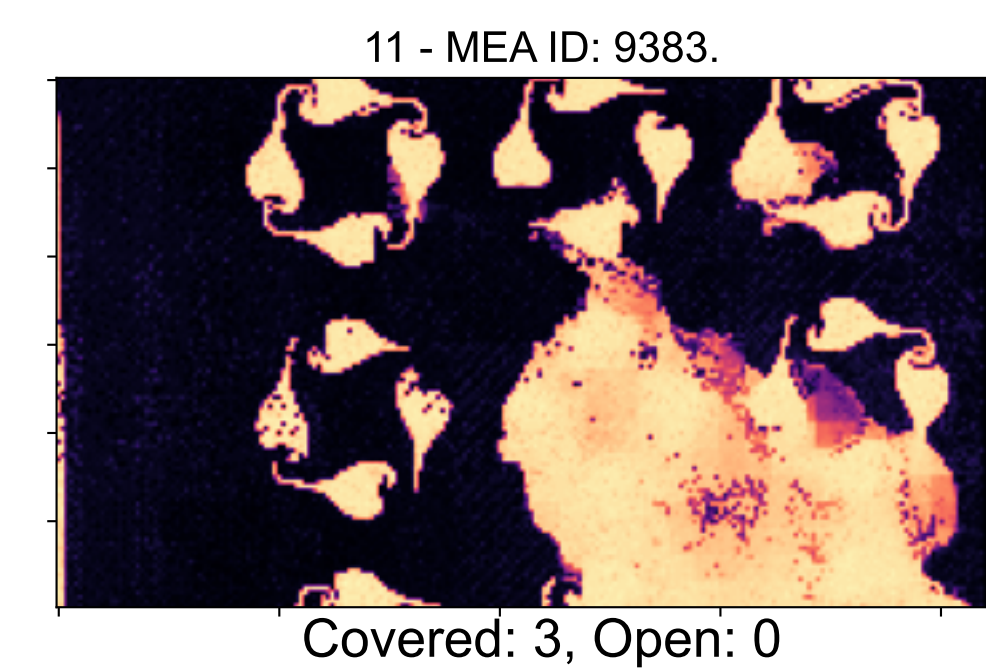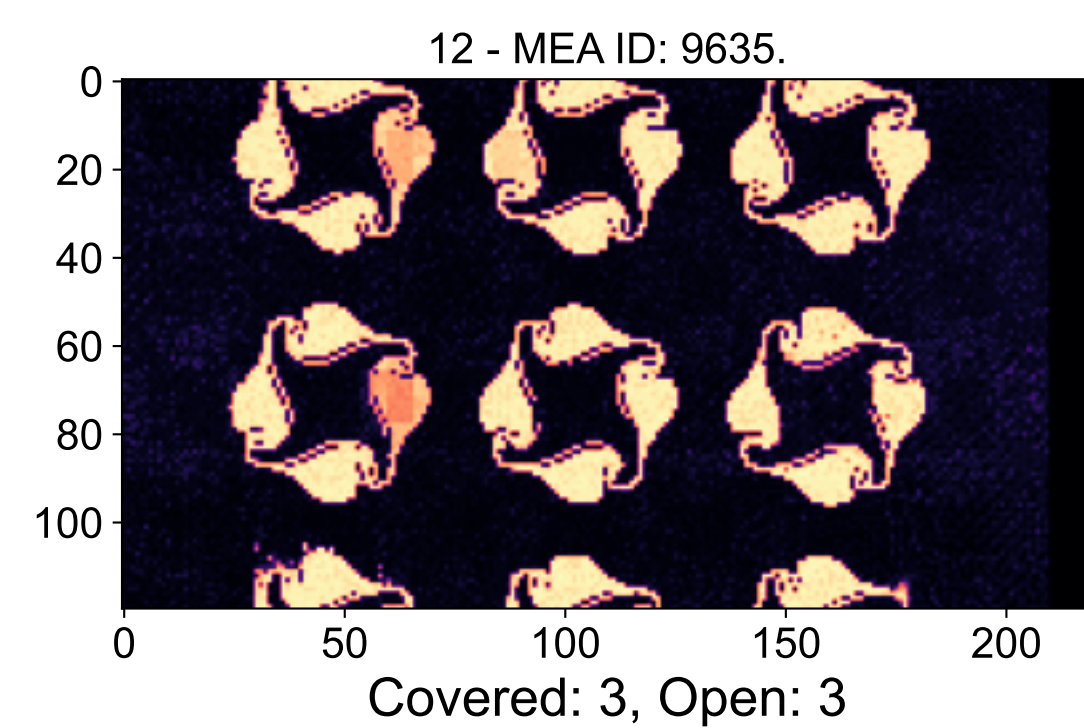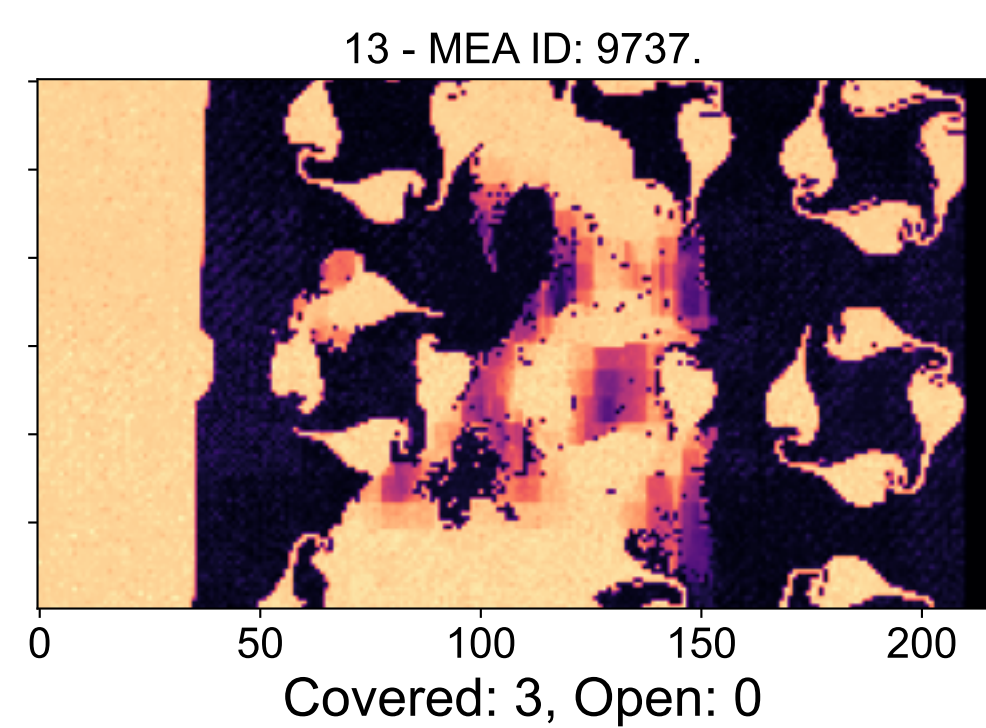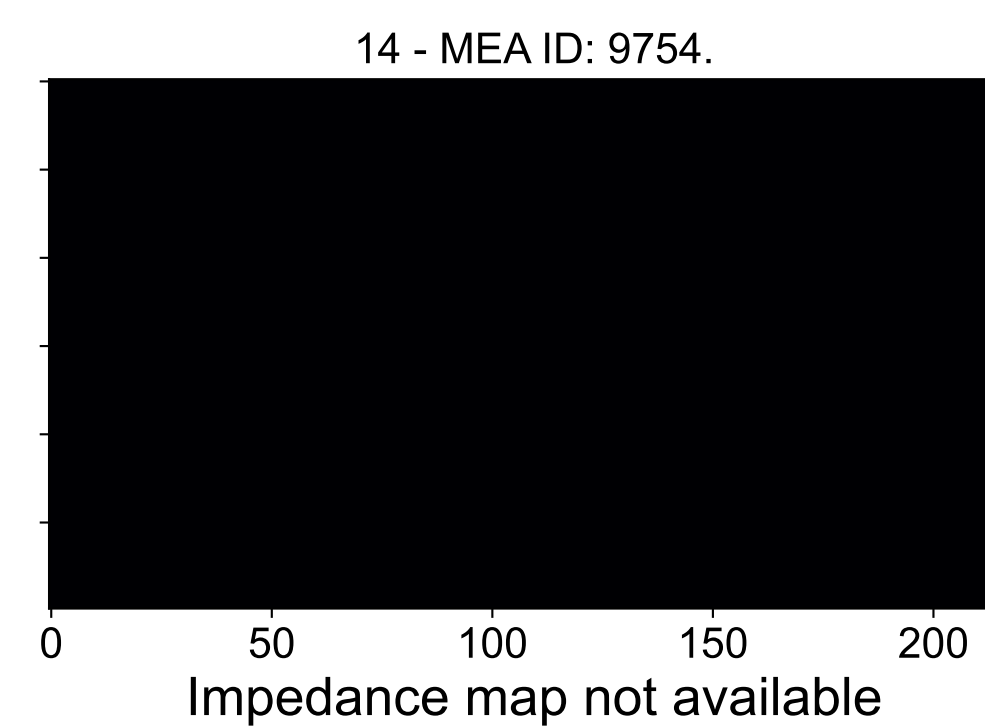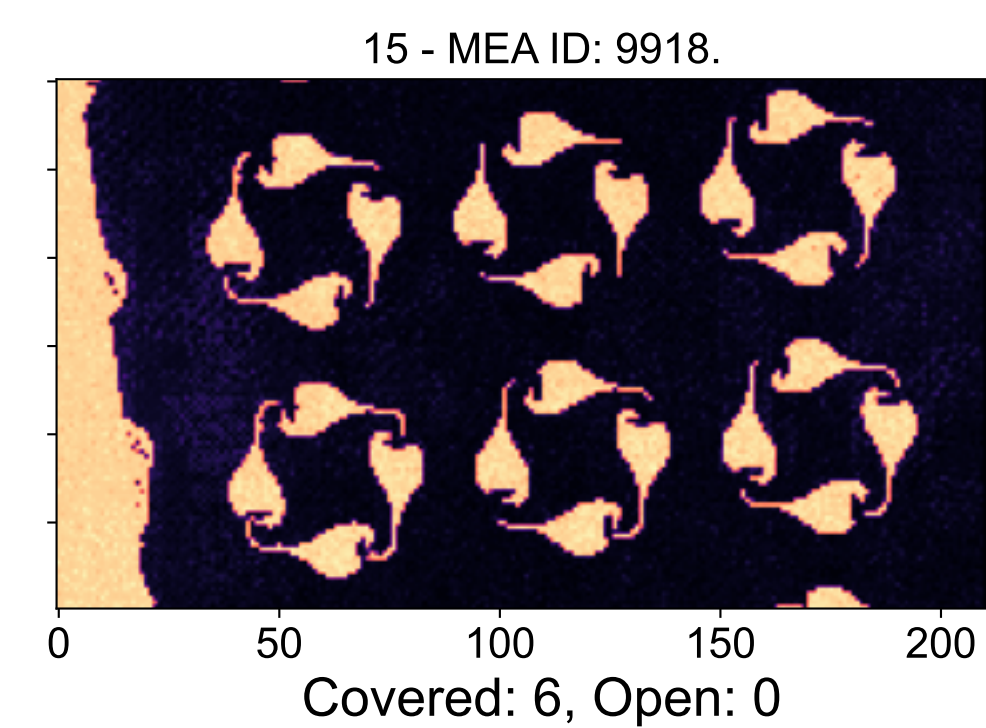

Supplement: Supplementary Datasheet 1 — Impedance maps of all 14 CMOS MEAs including information on how many circuits of the microstructures are entirely covered by underlying microelectrodes (“Covered”) and how many circuits are completely open (“Open,”) i.e., allow axonal ingrowth. [file Data_Sheet_1.PDF]
