# Supplementary material for: Engineered Biological Neural Networks on High Density CMOS Microelectrode Arrays
Source: Front Neurosci. 2022 Feb 21;16:829884. doi: 10.3389/fnins.2022.829884 (PMC8900719; doi:10.3389/fnins.2022.829884)
Supplement: Supplementary Datasheet 3 — SEM image of a recessed electrode on the CMOS chip surface with indicated projected trench height. [file Data_Sheet_3.PDF]

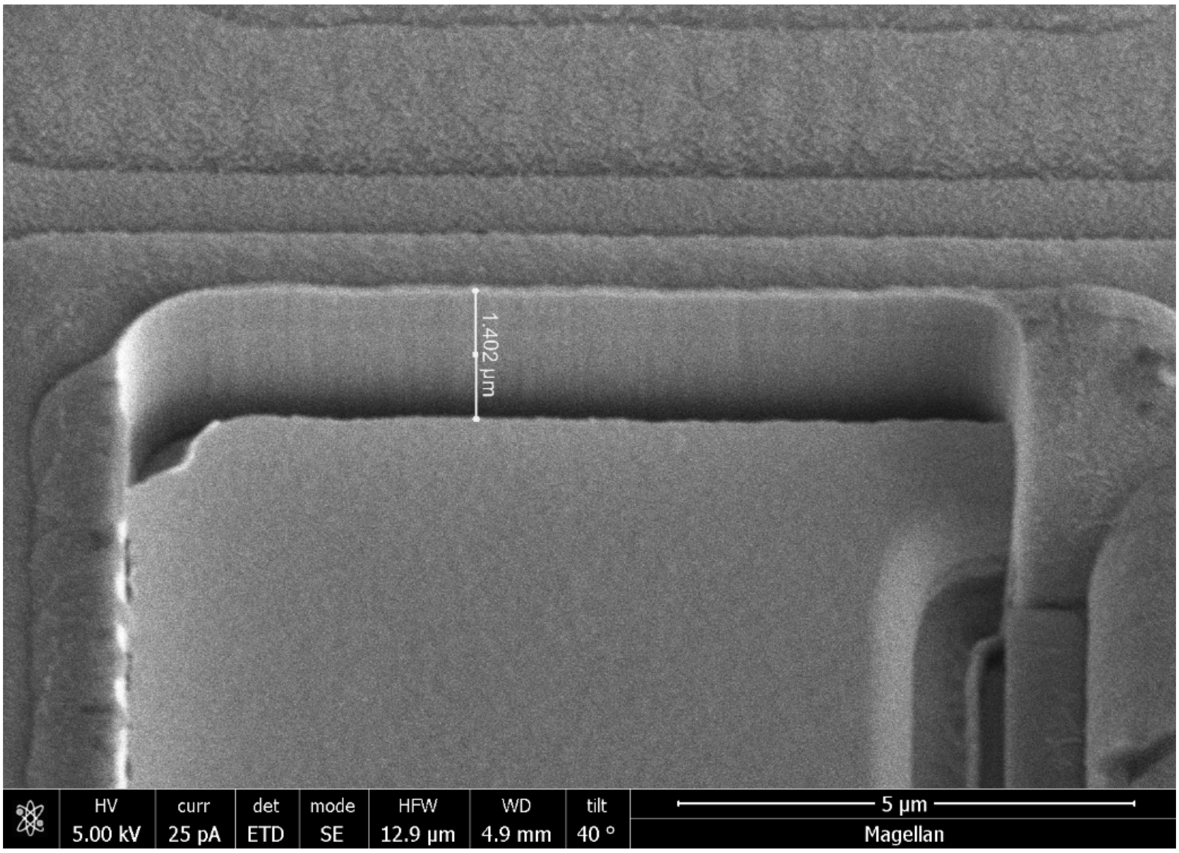

SEM image of a recessed electrode on the CMOS chip surface with indicated projected feature height h.

The figure above shows a measurement of the surface height of the CMOS chip. The height h was determined by acquiring an image under an angle of $\alpha = 40°$. The true height h_t of the well was calculated in the following way:

$$h_t = \frac{h}{\sin(\alpha)} = \frac{1.402\ \mu m}{\sin(40°)} = 2.18\ \mu m \tag{1}$$
