# Supplementary material for: Engineered Biological Neural Networks on High Density CMOS Microelectrode Arrays
Source: Front Neurosci. 2022 Feb 21;16:829884. doi: 10.3389/fnins.2022.829884 (PMC8900719; doi:10.3389/fnins.2022.829884)
Supplement: Supplementary Datasheet 4 — (A) Impedance map of the circular four node structures mounted onto the CMOS array. (Bi) Corresponding electrode blocks of the impedance map that were routed to generate the 23x23 electrode frequency map in (Bii). (Bii) Frequency heatmaps obtained by routing 23x23 electrode dense blocks around the microstructure channels. The observed activity pattern is clearly within the boundaries of the channel location when compared with the impedance map in panel (Bi). [file Data_Sheet_4.PDF]

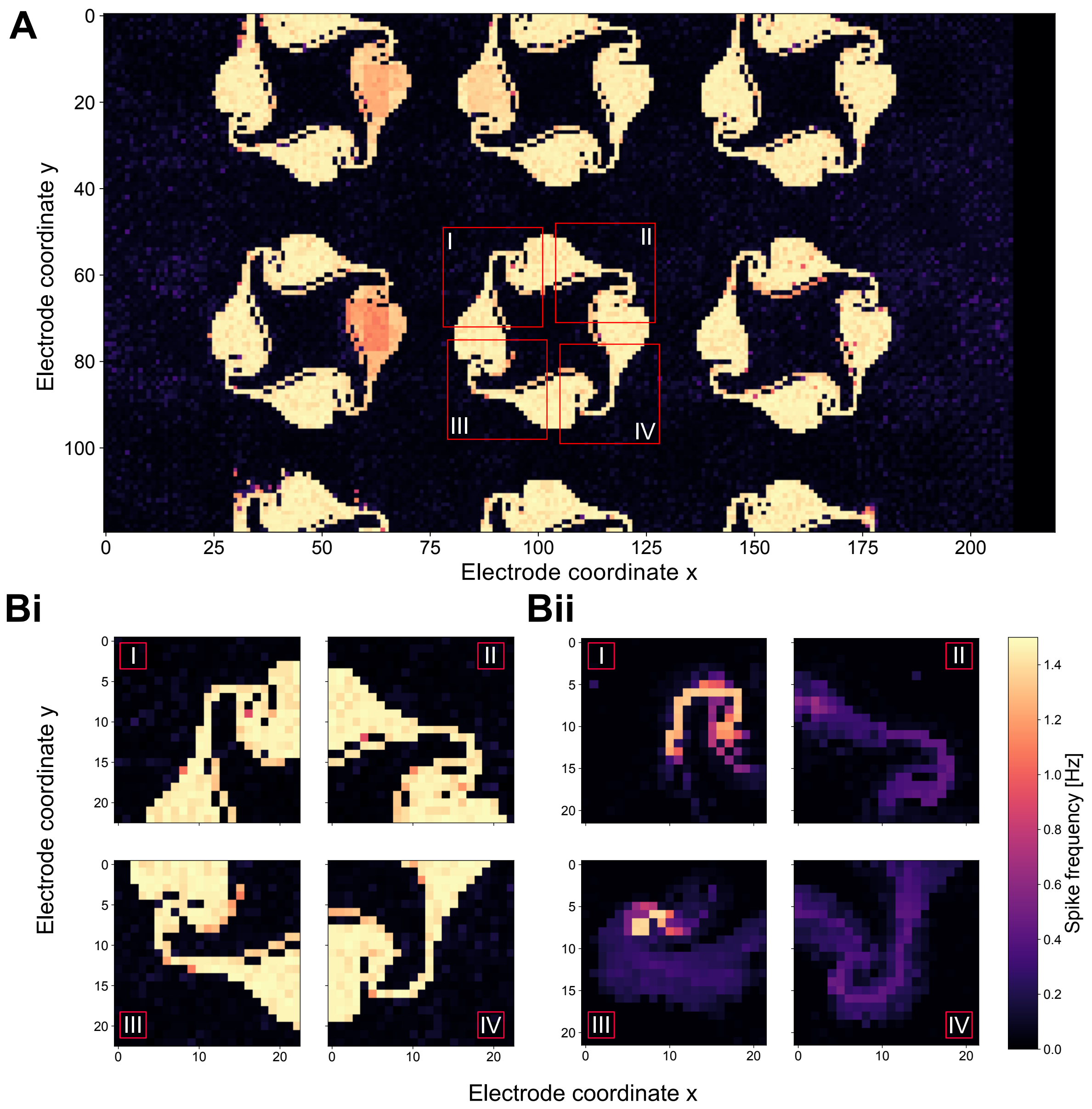

(A) Impedance map of the circular four node structures mounted onto the CMOS array. **(Bi)** Corresponding electrode blocks of the impedance map that were routed to generate the 23x23 electrode frequency map in **(Bii)**. **(Bii)** Frequency heatmaps obtained by routing 23x23 electrode dense blocks around the microstructure channels. The observed activity pattern is clearly within the boundaries of the channel location when compared with the impedance map in panel **(Bi)**.
