# Supplementary material for: Engineered Biological Neural Networks on High Density CMOS Microelectrode Arrays
Source: Front Neurosci. 2022 Feb 21;16:829884. doi: 10.3389/fnins.2022.829884 (PMC8900719; doi:10.3389/fnins.2022.829884)
Supplement: Supplementary Datasheet 5 — Assessment of PDMS microstructure adhesion to the CMOS chip surface. [file Data_Sheet_5.PDF]

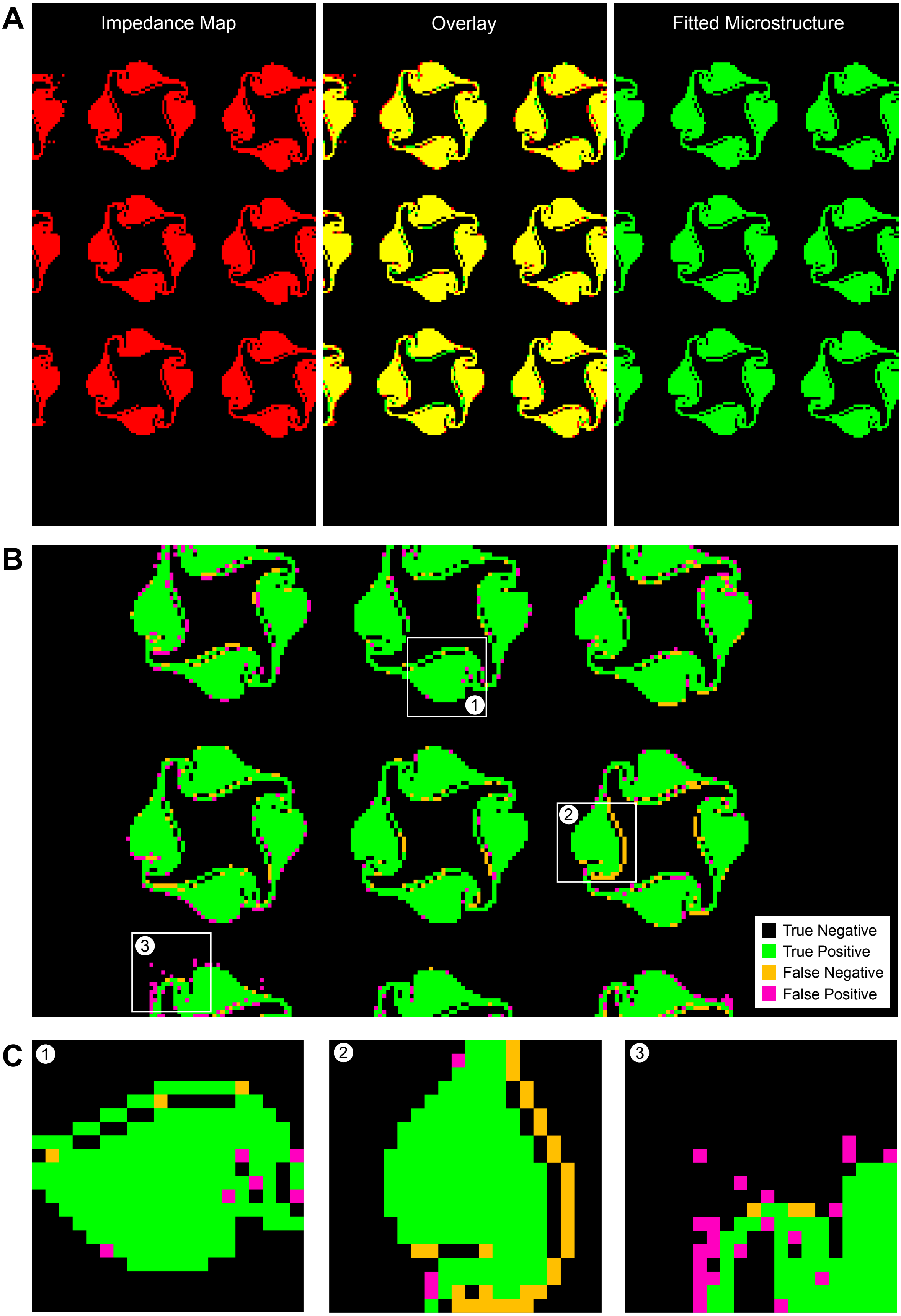

Figure 1: Assessment of PDMS microstructure adhesion to the CMOS chip surface. **A** *Impedance map:* Impedance map obtained after adhesion of the microstructure to chip 9635, *Fitted microstructure:* computationally generated image showing optimal mask transfer with no clogged or widened featuers. *Overlay:* Superposition of the impedance map with the optimal fitted map. **B**: Classification of every pixel (electrode) for sensitivity and specificity determination. **C**: Zoom into regions of interest of the impedance map.

In order to investigate the quality of the PDMS bonding step on the CMOS MEA surface, the impedance map shown in Fig. 3B of the manuscript was fitted to the known microstructures design consisting out of 9 circuits. The impedance map was transformed into a binary mask by thresholding. The threshold was chosen to be 0.5 times the maximal value of the impedance map. In order to align the microstructure with the impedance map, the following four parameters were fitted: a shift in the horizontal direction x, a shift in the vertical direction y, a rotation φ, and a scaling factor s. The alignment was achieved by using the following transformation matrix M:

$$M = \begin{bmatrix} s \cdot \cos(\varphi) & s \cdot \sin(\varphi) & x \\ -s \cdot \sin(\varphi) & s \cdot \cos(\varphi) & y \\ 0 & 0 & 1 \end{bmatrix} \tag{1}$$

The four unknowns in M were first estimated by hand until a rough alignment of the microstructure design on top of the whole impedance map was achieved. After this, the alignment was improved iteratively, by taking the currently best fit based on the F1 score and perturbing its four parameters with Gaussian noise. If the perturbed parameters yielded a higher F1 score than the previous set of parameters, the previous set of parameters was replaced with the perturbed parameters. In total, 1000 such iterative steps were performed.

Only a part of a CMOS MEA pixel consists of the sensing area which can detect electrical signals. Due to the rough surface area of the chip, it is difficult to know how large this area is. Therefore, it was heuristically estimated by maximizing the F1 score of the alignment. Based on this estimation, the sensor area has roughly a size of 7.7 µm × 12.6 µm. At the end of the fitting process, the F1 score of the whole alignment was estimated to be 0.938. Of the 26400 pixels (electrodes) of the impedance map, our algorithm showed that 20728 (78.52 %) electrodes were true negatives (covered by the microstructure and supposed to be covered), 5013 electrodes (18.99 %) were true positives (not covered by the microstructures and detected as such), 345 (1.31 %) electrodes were false positives (covered by the microstructure but detected as a non-covered electrode), and 314 (1.19 %) were false negatives (not covered by the microstructure but detected as a covered electrode).

In the following, we are going to investigate the 3 circuits in the center line of the impedance map shown in the above image, since these are the only circuits that are fully covered by pixels. The scores were obtained for each individual network separetely by cropping the voltage map around the center of each microstructure in a 50×50 pixel window. For these 3 circuits, we get an F1 score of 0.94±0.017, a precision of 95.4 %±1.8 %, and a recall of 92.7 %±2.4 %. The recall can be used to interpret the quality of the mask adhesion in terms of how many electrodes underlying the openings and channels of the microstructure are not clogged. In other words, on this chip 92.7 %±2.4 % of the electrodes that were supposed to be non-covered were indeed not covered.

Applying this algorithm to all 47 fully covered networks on all 14 CMOS MEAs, yields the results shown in Figure 2 of this document. Considering all of the networks, a recall of 77.4 %±9 %, a precision of 89.2 %±14.8 % and a F1 score of 0.81±0.08 is obtained.

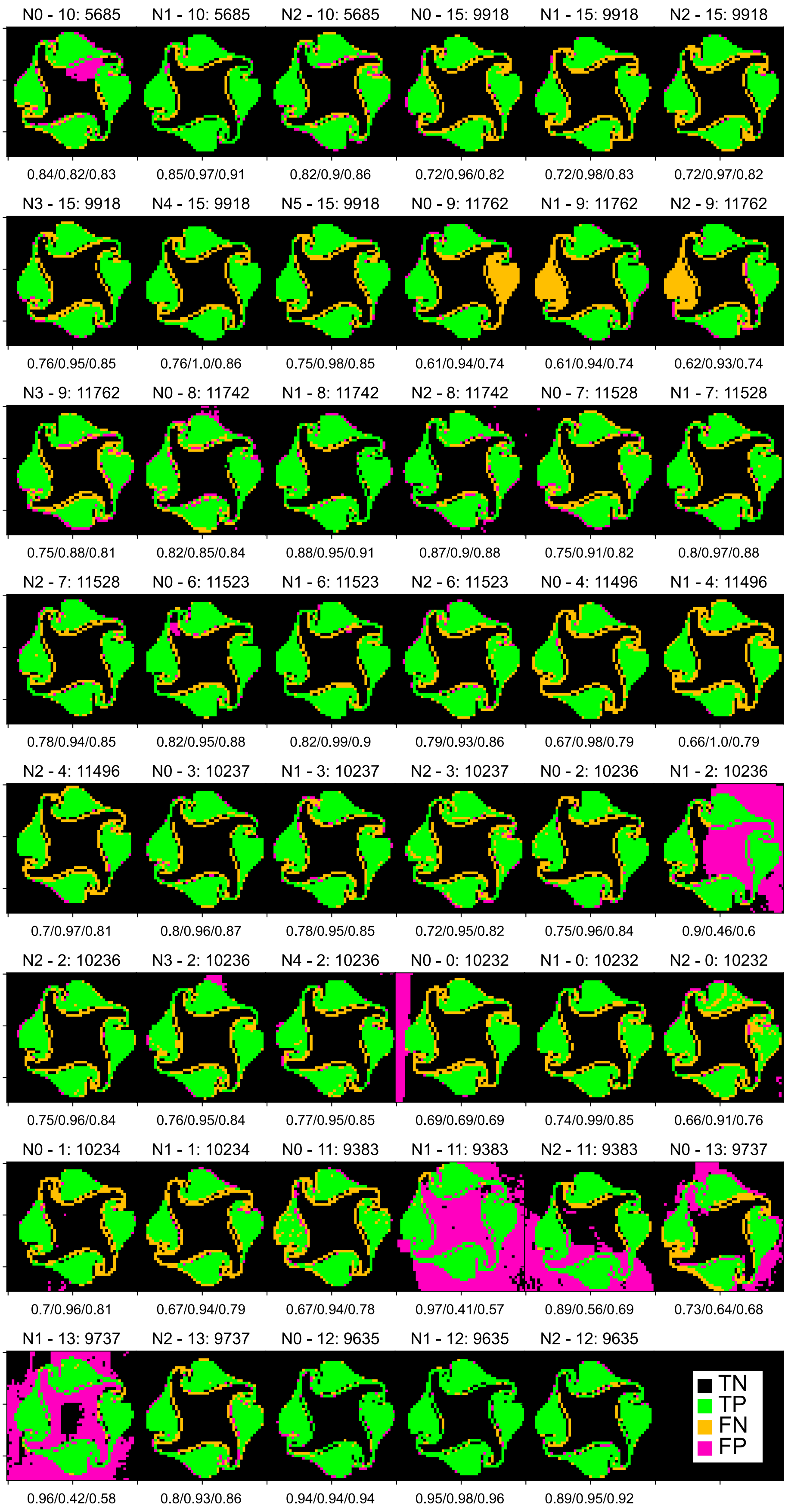

Figure 2: Assessment of the PDMS microstructure adhesion for all 47 networks. The title shows the network number, Chip ID and Number. The x-label shows the values for recall, precision and F1 score. Pink pixels: False positives (FP). Electrodes that were detected open but are covered by the microstructure, Orange pixels: False negatives (FN). Electrodes that were detected covered but are open, Black pixels: True negatives (TN). Electrodes that were detected covered and were indeed covered by the PDMS microstructure, Green pixels: True positives (TP). Electrodes that were detected open and were indeed open as determined by the PDMS microstructure matching the impedance map.
